# Five Vital Components in an Occupational Therapy-Based Municipal Weight Loss Program Obtained Through Research Circles

**DOI:** 10.3389/fresc.2022.822896

**Published:** 2022-06-10

**Authors:** Christina Jessen-Winge, Kim Lee, Signe Surrow, Jeanette R. Christensen

**Affiliations:** ^1^User Perspectives and Community-Based Interventions, Department of Public Health, University of Southern Denmark, Odense, Denmark; ^2^Department of Occupational Therapy, Institute of Physiotherapy and Occupational Therapy, University College Copenhagen, Copenhagen, Denmark; ^3^Department of Occupational Therapy, University College South, Esbjerg, Denmark; ^4^Research Unit for General Practice, Department of Public Health, University of Soutern Denmark, Odense, Denmark; ^5^Research Unit for General Practice, Aarhus University, Aarhus, Denmark

**Keywords:** obesity, overweight, danish obesity intervention trial, DO:IT, everyday life, rehabilitation

## Abstract

**Introduction:**

Currently 1.9 billion adults worldwide are estimated to be overweight or obese. Weight loss sustainability is difficult, and weight loss rehabilitation programs have been criticised for having an individualistic approach. It has been suggested that occupational therapists could be used as part of a transdisciplinary approach because of their holistic perspective. During the development of an occupational therapy- based weight loss program five components arose as essential from earlier interviews with health professionals and citizens with obesity: diet, physical activities, social relations, habits and balancing everyday life. Before developing the program, we needed a clearer picture of which strategies should support the five components. The aim of this study was to obtain a description of strategies related to the five components that could support weight loss to become part of everyday life of people with obesity.

**Methods:**

This study took a participatory design by using research circle (RC) methodology. Two RC groups were created, one with occupational therapists and one with citizens with obesity. Researchers participated in both RC groups. Data was obtained through democratic principles. The two RC groups met four times over a period of 4 months. Qualitative content analysis was conducted.

**Results:**

Five themes arose: (1) Diet—Find the line between either-or, (2) Physical activity—Break the comfort zone, (3) Social relations—Stand strong together, (4) Habits—Focus on possibilities instead of bad habits, and (5) Balancing everyday life—Handling life's bumps.

**Conclusion:**

The strategies for a weight loss program including the five components should include awareness of senses and activity patterns surrounding meals, taking one step at a time when focusing of physical activities and be conscious of values, include family and friends in the program to find common ground and self-confidence and use re-placement activities. We suggest that the component of balancing everyday life should be seen as an overall component as all strategies are related to finding meaning and variation in activities which is part of an ongoing weight loss process.

## Introduction

Obesity has been labelled “the greatest public health challenge of the century,” and has been acknowledge as a chronic disease by the World Health Organization (1). The prevalence of people living with overweight or obesity was 1.9 billion and of those, 650 million were obese in 2016 (2). This is a challenge because living with obesity affects a broad spectrum of life (3). Obesity is associated with health conditions such as cardiovascular disease, cancer and diabetes mellitus (2). Moreover, physical and psychological consequences of obesity (e.g., pain and feelings of being less valued) have an adverse impact on everyday life understood as components of activities and participation (4–6). Activities such as playing with children, buying clothes and going for a swim that are taken for granted were found to be adversely impacted by obesity (3).

Rehabilitation programs that combine physical activity, diet, and behavioural therapy can result in significant weight loss (7, 8). However, maintaining weight loss remains elusive (7). Some have argued that existing programs have failed because they treat the process as a problem of individual agency that is addressed outside of the individual's everyday life (9). This approach stands in contrast to an occupational therapy perspective advocating for a more holistic approach (10). The holistic approach within occupational therapy attend to the link between person, activities and the environment and how these factors all influence participation in everyday life (11, 12).

The Danish National Board of Health recommend that weight loss programs should include a combination of diet exercise, behavioural therapy and everyday life (13). This is further detailed in earlier studies with the aim to understand wishes for the content of weight loss program both from the health professionals and the citizens with obesity (descripted only as citizens in the following). The studies, based on 66 individual interviews, showed that both groups wanted to focus on meaningfulness rather than narrow components like sports and calories, and that it should be implemented in everyday life if sustained weight loss and increased well-being were to be realistic outcomes. The two groups agreed that five components of a trustworthy program should be diet, physical activities, social relations, habits and balancing everyday life (14, 15).

Obesity is a complex phenomenon wish needs to be addressed from a transdisciplinary view. Health professionals conducting weight loss programs should be medical doctors, dietarians, physical therapists, nurses, psychologists and occupational therapists (13). Despite the recommendation and potential benefit of applying an occupational therapy perspective to a transdisciplinary weight loss rehabilitation program with their focus on participation in everyday life, the research is sparse (10). To address this knowledge gap, we wish to develop an occupational therapy-based weight loss program as an ad-on to existing weight loss programs for municipalities. The municipalities in Denmark are responsible for conducting weight loss programs for its citizens. One of our earlier studies, a survey of all weight loss programs in the municipalities, showed that the programs primarily included one or two health professionals (most often dietarians) and that the programs were heterogeneous in length and content (16).

In the developing phase of the occupational therapy-based ad-on weight loss program we wanted to understand the role of occupational therapists more deeply. This was done by understanding strategies that could support the five components from the view of occupational therapists. As the focus for occupational therapists are the everyday life of citizens the perspective from occupational therapists should be understood in relation to the perspective of the citizens. Therefore, the aim of this study was to obtain a description of strategies related to the five components that could support weight loss to become part of everyday life of people with obesity from the perspective of occupational therapists and citizens.

## Materials and Methods

### Research Design

Based on the roots of Gadamer's hermeneutic understanding this study took a participatory design by using a research circle (RC) methodology (17). The methodology emphasises collaborative cooperation between researchers and participants to develop knowledge through discussions and reflections. We applied the principles of RC methodology with the aim of discussing strategies relating to the five components obtained from the previously conducted 66 interviews (14, 15). In accordance with the Danish National Committee of Health Research Ethics, ethical approval is not required for studies that include only interviews.

### Participants

Participants were recruited to two different RC groups representing occupational therapists and citizens. The researchers who participated included a PhD student and two master's degree students were all occupational therapists. They had the role of moderators. The target sample size was five – eight participants for each group to make sure that everybody would be heard (18).

A purposeful sampling approach was used. Citizens were included if they self-reported a BMI +30 and had experiences with weight loss attempts. Exclusion criteria was if the citizens did not speak or write Danish. The citizens were selected from waiting lists for weight loss programs offered in one of the Danish municipalities. Health professionals from the municipalities helped the research team by phoning citizens on the waiting lists and presenting the study information to potentially interested citizens. We introduced the health professionals to the project by phone and thereafter emailed them a detailed description which they used to inform the citizens.

In our previous finding content for a transdisciplinary collaboration was included, however specific knowledge about the role of occupational therapists in the collaboration seems to be missing (15). Inclusion criteria for the occupational therapists were that they had worked with group-based weight loss rehabilitation programs in a municipal setting at least three times before. To ensure that the focus was only weight loss, programs combining weight loss and a diagnose such as diabetes were excluded. They were invited by a snowball method as we contacted occupational therapists which we knew could be included and they helped the further process.

Signed informed consents were obtained from all the participants who agreed to participate in the project in accordance with the Declaration of Helsinki.

### Data Collection

The two RC groups met four times in the period from February to May 2020. The meetings were planned to be conducted every third week in a meeting room at the University of Southern Denmark. However, because of the Covid-19 pandemic the last two meetings with each group were virtual Zoom meetings. Each meeting lasted 2 h. Diet and physical activity were discussed in the first meeting, habits and social relations in the second, and balancing everyday life and sustainability in the third. In the fourth meeting, we validated the preliminary results. All meetings started with a presentation given by one of the researchers, based on the knowledge from previous studies (14–16, 19) and meetings 2–4 also included a summing up of the previous meeting, with time for comments and reflections. Activities and debate questions to facilitate discussions and secure equal participation were prepared for all four meetings (Table 1). The researcher had the role as facilitators, however in accordance with the RC democratic principles knowledge from all participants was discussed on equal terms (18). All meetings were conducted in Danish, audio recorded and transcribed into 312 pages. All data were stored in a safe database provided by the University of Southern Denmark and all participants were anonymised in the transcript material.

**Table 1:** Procedure of the research meetings.

	**First meeting**	**Second meeting**	**Third meeting**	**Forth meeting**
Content	Diet and physical activities	Habits and social relations	Occupational balance and sustainability	Validation
Procedure	Presentation of results from previous studies	Validation of manifest content from the first meeting	Validation of manifest content from first meeting	Presentation of results from the previous meetings
	Recommendations about diet and physical activities	Presentation of results from previous studies	Presentation of results from previous studies	Discussion and validation
	Working in pair with questions and reflections about how the components should be part of a municipal weight loss program	Presentation of research of habits and social relations related to weight loss	Presentation of recommendations about weight loss and weight loss maintenance	Evaluation of the entire process including all four meetings
	Supporting and building on each other's ideas by saying “Yes-and-then” Evaluation of this day's research circle	Discussion about habits Video sequences of a couple talking about how they support each in doing nothing Discussion about the video and social relations Evaluation of this day's research circle	Theory about occupational balance Discussions about the content Evaluation of this day's research circle	

### Data Analysis

The data was analysed using qualitative content analysis on a manifest level between the first three meetings separately for each RC group (20). This analysis consisted of four steps. In the first step, all the data material was read by the researchers. In the second step, content relating to the component of the meetings was identified and divided into meaning units. In the third step, the meaning units were condensed and labelled with a code. The codes were then aggregated into subcategories and categories. Between the third and fourth meeting, the categories from each RC groups were discussed between the researchers to be merged into 12 final set of categories. The 12 categories were discussed with the participants for validation in the fourth meeting. Knowledge from the last meeting was discussed between the first two authors and the master's degree students in order to refine and modify the merged categories until five themes emerged which seemed to fit the data in the best possible way (see Table 2).

**Table 2:** Themes and categories.

	**Physical activities**	**Diet**	**Social relation**	**Habits**	**Occupational balance**
Themes	“*Break the comfort zone by finding positive value”*	“*Find the line between either or”*	“*Stand strong together”*	“*Focus on possibilities instead of bad habits”*	“*Handling the bump in life with adaptability”*
Merged categories	Everyday life instead of recommendation Crossing boarders slow through doings Find physically, social and mental values and rewards	From restrictions to enjoyment Easy to relate to everyday life Focusing on culture regarding food Conscience about actions and feelings before changing	Focus on secondary relations both under and after weight loss The family both influence and are influenced Strategies to meet the world	Strategies into the everyday life in a never-ending story Learn to stand strong alone The context is a central part of the changes	Weight loss should be one activity goal out of many Variable structure Raise self-conscience through experience and meaningfulness
Categories Citizens Group	Use the opportunities at home Involve other Do not follow the recommendations	No restrictions Relate it to everyday life Focus on the feelings	Managing conflicts in social connections Information to nearest network to stop negative support Support after the program for sustainability	Fighting the bad habits Implementation in everyday life Repeating small changes Record changes	Balance in activity goals Increase self-conscience through different activities Find structure through doings
Categories occupational therapist Group	Start where they are Cross boarders Focus on values	Senses instead of restrictions Consciousness about eating habits Practical implementation	Navigate in a mutual influence Use relations for sustainability Different needs	Learn to stand strong Handling an ongoing process The context is part of the habits	Life is in focus, weight loss is a positive side effect Experiences should be the starting point Small variations are the way to success

## Results

Thirteen participants were represented: three citizens, seven occupational therapists and three researchers (Table 3). In the findings, the citizens will be represented with a C and a number and the occupational therapists with an O and a number.

**Table 3:** Participants included in the study.

**Participants**	**Gender**	**Age**	**Employment**	**Part of meeting**
**Citizens**				
C1	F	34	Unemployed	1, 2, 3, 4
C2	F	64	Senior citizens	1, 2
C3	F	52	Healthcare worker	2, 3, 4
**Occupational therapists**				
O1	F	34	Health centre	1, 2, 3, 4
O2	F	42	Health centre	1, 2, 3, 4
O3	F	42	Occupational therapist education	1
O4	F	32	Day offer for young people	1, 2
O5	F	41	Health centre	1, 2
O6	F	48	Privat company	2, 3, 4
O7	F	31	Health centre	3, 4
**Researchers**				
R1	F	27	Master student	1, 2
R2	F	25	Master student	1, 2
R3	F	47	PhD student	1, 2, 3, 4

### Diet—Find the Line Between Either-Or

The two most significant strategies discussed was addressing consciousness and satisfying the senses. One central element from both RC groups when discussing consciousness was that prohibition and registration of what to eat should be avoided. O1 stated this very clearly by saying:

*O1: “it is very, very important that it doesn't become a prohibition. Above all, it must not be a prohibition. Now you must* *never* *ever eat cake, or you may never again eat cake.”*

C2 agreed that there was nothing called forbidden food. She explained that if she failed to eat healthy when following a diet, she felt bad and suffered from a bad conscience.

However, all the participants agreed that the path to taking a decision without bad conscience was difficult. According to O3 consciousness should be the first step:

*O3: “I think it's got something to do with – as far as citizens are concerned – it has something do to with consciousness of the choices they make before they change them.”*

The occupational therapists said that the way for the citizens to realize what and how much they ate should be by asking about their everyday life. O2 gives an example:

*O2: “I can see that when I ask them about their diet, they say but I don't eat anything, and they turn up and have a BMI of 49. But I don't eat anything […….]. And you get the urge to say: ‘**How*'*s that?*' *and you don't of course but you get the urge to say well now, please listen carefully, what exactly is that's happening?.… Well, then you hear them say: ‘Well, I love rice'. Well okay then [….] Well and in that way, you start to get the true picture. So, I also think there's lots of things like that; asking and narrowing it down with the different things in everyday life.”*

The participants connected consciousness to the patterns surrounding food. O3 pointed at the shopping pattern, while C3 pointed at the eating pattern and explained that her eating patterns was unsuitable because she ate in front of the TV, but she didn't know how to change it. C1 suggested using a sort of diary to write down what happened:

*C1: “…get some kind of form you can use at home so you can write it down there. In other words, I become aware of when I do something. And perhaps you do it simply by writing it down, on an ongoing basis.”*

The citizens also connected consciousness to senses. C1 said that instead of just “eating with the eyes” one should use several senses. The occupational therapists believed that changing the line between either or should be found in the senses like taste, colour and odour. Senses should be used instead of talked about. O2 said:

*O2: “There is something about the senses that might work. I mean all the stuff about sensing and especially tasting. “*

### Physical Activity—Break the Comfort Zone

All the participants agreed that being obese made it unrealistic to follow recommendations from health authorities about spending time being physical active. Nevertheless, all of them found it important to carry out more activities in everyday life by using the strategies small steps and value. The citizens pointed at integrating physical activity with activities done at home instead of going to the fitness centre. C2 explained how she structured doing her laundry to get more physical activities:

*C2: “It's because we live on the first floor. I've organised it so that when I fold clothes, I fold piles with tops, piles with socks and piles with trousers, etc. Then I carry each pile up. That way I don't carry everything in one go. Before, I would put every pile in a single basket and carry the whole lot up.”*

O5 said that instead of giving a lecture about recommendations they should talk about all the things they already do at home, like vacuuming or gardening. C1 stated that understanding physical activity as home activities made it more manageable. However, doing things at home did not make up for the lack of physical activity in the long run. The occupational therapists especially emphasised the importance of leaving one's comfort zone. O2 explained that it is important to start with small steps and then to build on the process:

*O2: “I completely agree that they need to start on a small scale and build things up from there, so that they are not frightened off. But at the same time, making sure they are not in their comfort zone all the time. Otherwise they will go back to doing what they usually do.”*

Crossing new lines, small victories was experienced. O1 recommended bringing the citizens to sports clubs to overcome practical barriers like going to the dressing room, overcoming the feeling that your heart is beating too quickly and to get introduced to different sports. This gave the citizens the opportunity to find value with physical activity. Values were highlighted in both RC groups. O5 explained that the value could be both physical, psychological and social but the point was to make the citizens realize there were different possibilities with physical activity. O2 suggested using narratives:

*O2: “….what things did you do when you were young? And what did you do during your active adult life? Well now, three years ago myself and my husband would go dancing and we don't do that anymore. Well then, why don't you start that again?”*

### Social Relations—Stand Strong Together

The strategies that were emphasised were self-confidence and common ground. Both RC groups divided social relations into two groups; a primary group, which was families and a secondary group, which was friends, peers and health professionals. The primary social relations are more complex than the secondary group. Peers seemed important because of the possibilities of including new activities in everyday life and by exchanging experiences. For the citizens, professional advice was stated as essential. C1 wanted advice when starting something new or felt physical pain and suggested Facebook to get prolonged advice. The occupational therapists used Facebook but seemed sceptical about a prolonged advice period because they pointed out the importance by learning to handle changes and stand strong alone. As stated, the primary group's social relations are more complex because changes here might affect the whole family. O6 puts it like this:

*O6: “You must never underestimate how often it can create major conflicts if you go in and set up some goals or create major changes in a family structure.”*

According to O5, the citizens should handle these potential conflicts as they should decide what changes are necessary to reach their goals. It could be major changes, for example divorce or minor changes, for example, cooking food differently. O2 said that taking the power boosts self-confidence:

*O2: “You can certainly involve close family members in one way or another but for some people being able themselves to come home and say ‘You know what? We can do things this way' – it's a real boost. So, they themselves reclaim power and so you can say that it isn't us who have the power.”*

The most important thing for C1 was to include her partner in the program to find common ground. She wanted him to understand how her overweight made her feel physically and psychologically deprived. She explained how she had experienced that her partner opposed her project if he did not understand it or did not find the changes important. C3 found it difficult to integrate a partner but very important for avoiding a conflict. She worried how life could be if they should eat different food or even eat separately at different times or places.

### Habits—Focus on Possibilities Instead of Bad Habits

Habits seemed essential with strategies turning to resources and action. The citizens first reaction on this subject were bad habits. C1 simply said:

*C1: “Well now, I also quickly think bad habits. And I think it's slightly because the thing that* *I* *wrestle the most with, is bad habits.”*

The occupational therapists found working with bad habits important because this “*fight against bad habits*” was attractional and led to negative feelings about their ability to change. O6 used a metaphor as a backpack filled with good and bad experiences, but the only thing in focus were the bad experiences.

*O3: “But because they have completely succumbed to “I'm no good for anything. I can't do anything, and I gain weight every time I drive past the bakery!”*

Both RC groups highlighted the need to change these feelings to achieve positive attitude and success. Overall, the occupational therapists expressed the importance of using conversations to find resources there could bridge to habit changes, while the citizens focused on carrying out activities. The OTs used positive thinking to break the vicious circles, dreams to understand feelings and resources from earlier successful attempts to change habits. Talking about feelings and resources was done to show how to transfer these actions to the next action. O2 explained:

*O2: “Is there a way you can find calmness in some other way rather than eating four Victoria sponge cakes? I get to grips with the feelings and find out what it is that you get when you carry out this action even when you don't want to do it because it's you who carries out the action and you do it because you get something out of it. Well okay. It gives me calmness, or it gives me pleasure or it gives me something or other. It comforts me. Okay, can you call someone and get comfort that way instead of.…”*

The citizens agreed that feelings and habits were connected because they often felt sorry for themselves. However, they all stated that carrying out activities was the way to achieve success. C2 said that anything is better than the couch and even carrying out minor activities gives you energy to move on. C3 followed this by explaining how activities can lead to positive thinking:

*C3: (Smiling) “That's precisely how it works. And then they gradually realise: ‘God. I bloody well have some backbone.…”*

### Balancing Everyday Life—Handling Life's Bumps

Weight loss was understood as a never-ending process incorporated into a changeable life with bumps along the way. This should be handled with the strategies of finding meaning and including variation throughout everyday life. Bumps were explained as multiple situations such as starting to gain weight again, having an argument with a friend, divorce or being fired. Often, the way this was handled was by eating or going back to the couch. The central way to change this is to find meaning and incorporate it into everyday life:

*O4: “The phrase that pops into my head is actually meaningful everyday life. That it should be meaningful for the individual person to make these changes and carry out these initiatives. Not just do them. But that they should have meaning and build further on them.”*

Meaning was experienced differently by C1 and C3. C1 found weight loss to be meaningful because she was in a place where she had to lose weight to do the things she wanted to do. Conversely, C3 had accepted her weight, and was focusing on walking long distances because she loved doing it. She explained the importance of being adaptable in the process:

C3: “… *you must be very open to change, and I have to say that's the first thing I have learned after having walked on the pilgrimage. It helps that changes have happened and at last I could be part of this. And it was a long time before I finally understood that you have to be willing to change*.”

Even though C1 found weight loss meaningful in her life, she wanted her activities during the day to have different meanings. She knew this could be difficult:

*C1: “And that's something I'm working on, sometimes you have to leave off thinking I'm doing this because I have to maintain my weight, or I have to lose weight. I take the dog for a walk simply because I think walking the dog is really enjoyable.”*

This quote indicates that being open to change was linked with variation in everyday life. Achieving variation and finding meaning was understood to be a difficult process which could be supported by structuring the days for a period. The occupational therapists thought that a flexible structure could be part of a weight loss program, but having too much structure is the same as overly focusing on diet again:

*O2: “Well, I believe that structure can provide meaning. But you can also have too much structure, if you can put it that way. So, you become a slave to structure. You're helpless without it. No. Now maybe it's too…… well that is, you can easily have a structure from the beginning, where you say okay for the first three weeks, since I want to try to do things this way but in my world, you need to have it as a learning experience. Okay. It worked for me or it didn't work for me. So, in terms of structure for structure's sake, I only have a little remaining, relating to diet.”*

## Discussion

The aim of this present study was to obtain a description of strategies related to the five components and how they could support an occupational weight loss rehabilitation program. The participants found it feasible to work with all five components connecting several strategies to them: For diet, the strategies were senses and consciousness, for physical activities, values and small steps, for social relations, self-confidence and common ground, for habits, resources and actions and for balancing everyday life, meaningfulness and variation. In the following the strategies will be discussed and specified.

### Diet—Find the Line Between Either-Or

The participants found that strict diets were unsustainable as it made the citizens feel that they were either trying to lose weight or not. Therefore, it was pointed out that prohibition and registration of what to eat should be avoided when changing eating behaviours. Instead, they focused on awareness related to activities and senses surrounding the food. This invites to a more holistic approach understood as involving both the person wanting to change and activities (10).

More specific the occupational therapists connected the person and the activity of eating by focusing on the senses. They highlighted that they met citizens who often ate without being aware about what they ate or how it tasted. To enable the citizens to stick with a diet conductive with weight loss the occupational therapists believed a strategy in weight loss programs should be encouraging awareness of the specific activities of eating by the utilisation of the sense such as taste, smell, texture, colours, and sound. This strategy is in line with a successful non-diet program called “Health at every size” valuing eating by empowering citizens to eat based on hunger, nutritional needs and pleasure instead of regulations. In this programs the importance of nutrition topics are addressed for weight loss (21). This is an important example of how the strategies from an occupational therapy perspective should be connected to knowledge from other professionals about nutrition in a transdisciplinary collaboration.

However, according to the participants the activity of eating should not only be connected to the person and the senses but also to an understanding of the patterns of activities related to eating both before, during and after, e.g., a shopping pattern or the pattern of watching TV during eating. Patterns are seen as a regular way of acting or doing something and therefore an activity pattern could be understood as activities performed during a day, a month, a year or a life (22). Activities during everyday life influence each other in a dynamic system like every time I watch TV, I eat chocolate. Therefore, to understand the patterns we suggest that citizens wanting to lose weight should write down what they do in relation to the activity of eating as suggested by one of the citizens in this study.

### Physical Activity—Break the Comfort Zone

The overall goal with this component according to the participants is to support citizens with obesity integrating more physical activity in everyday life. The literature describes physical activity as an important component for losing weight and recommendations vary from 20 to 30 min five times a week, to 1 h every day (23). These recommendations have been criticised for not being feasible because of physical limitations caused by obesity (24). The same understanding was seen from the participants as they meant that these recommendations were unrealistic. They pointed at two strategies for integrating more physical activity: taking small steps and finding a way around individual values.

The Danish National Board of Health recommend implementing small steps in any weight loss program (25). The occupational therapists in our study found this recommendation useful, especially in relation to changing physical activity. Small steps could be understood as part of an activity analysis with the purpose of understanding tasks and environment related to physical activities (26). This analysis should result in finding the right level to start and then building opon this knowledge. This strategy was explained by our participants as they recommended increasing physical activities in the home environment, by extending activities which were already implemented in everyday life.

However, to implement sustainable physical activities in everyday life the participants pointed at the importance of values. Focusing on values are in line with a study from Toft et al. (27) describing individuals need to find the way around physical activities based on values and possibilities instead of “drifting along with others' choices of living” (27). Values are individual and need to be developed though discussions and experiences (27). To support the discussion the citizens need to be aware of earlier experiences with physical activities like suggested by one of the participants in this study. This could be done by telling the story of physical activities and its value throughout a lifetime. This method has been developed by Clark et al. and is named “activity—story—telling” (28). By combining “activity—story- telling” with experiences though doing physical activities in e.g., the municipality setting, the telling could be turned to “activity—story—making.” Combining the telling with experiences could then turn to “activity story making” with new perspectives on values and meaning and might then be transformed to a future story including physical activities (28).

### Social Relations—Stand Strong Together

It has been pointed out that group-based weight loss programs are more effective than individual programs because of the relationships between participants, peers and health professionals (29). In our results, these social relations are called secondary relations and seems easier to include compared with primary relations. The primary relations were understood as complex but interestingly, there seemed to be a discrepancy in how to handle this complexity. The occupational therapists highlighted the importance of standing strong to achieve confidence, while citizens found it important to integrate the changes with the nearest network. Learning to stand strong by working with self-confidence and self-efficacy could be done through behavioural therapy, which is recommended in weight loss rehabilitation programs (30). This perspective is related to an individual approach, where every person has the opportunity to fix the problem by themselves (11).

The citizens perspective showed that the social relations need to go beyond support. Family members or close friends should do things together with common value and meaning in the activities they engage in during a weight loss transition. Finding common value and meaning in activities could be explained as co-activities describing activities done together more than one person with shared physicality, emotionality and intentionality (31). To implement this and support sustainability family and friends need to be seen as an important part of a weight loss process because citizens are embedded in and influenced by the social context of their everyday life (11).

### Changing Habits—Focus on Possibilities Instead of Bad Habits

The participants talked about bad habits pointing out that the negative things in their life influenced their belief in achieve weight loss. The strategies recommended by the participants were focusing on possibility through their resources and by doing activities. This is in line with the thoughts from Dewey about learning-by-doing. According to Dewey, the connection between one's activities and the consequences becomes, with experience, embodied within the person as habits. The habits will be deeply rooted by doing the activity related to the habit (32). Habits are complex because they contain several levels. Gardner (33) distinguish between (automatic) initiation and (conscious) performance of behaviour and suggests that one activity will include both, e.g., going to the gym might be a conscious decision, while exercising at the gym and showering afterwards might be an automatic action (33). Habits are by this understanding operating below conscious and are triggered by environmental cues. Implementation intervention has been suggested as one way to develop new habits (34). Implementation intervention could be described as re-placements-activity and has been shown to be successful in weight loss maintenance (14, 35).

We thereby suggest that replacements-activities should be part of the strategy of using the citizens resources and activities to understand and navigate in the complexity of habits.

### Balancing Everyday Life—Handling Life's Bumps

Weight loss and weight loss sustainability are often seen as two different phases, with the sustainability phase understood as an extended care model (7). One way of going through these phases is by structuring everyday life by doing the same activities at the same time almost everyday day (14, 15). This is extended in this study by using the word “a flexible structure” including words like variation and meaning indicating an integration of the two phases. A similar approach is recommended by Greaved et al. (36) who suggested that phycological strategies like motivation and self-esteem should be included in both weight loss and weight loss sustainability. The participants in our study wanted weight loss to be part of everyday life, however not all activities should be related to weight loss. The activities related to weight loss should be part of a variated life filled with activities having other meanings and purposes. However, participating in for example physical activities could change meaning along the way, like the story from the citizens about how taking a walk could change meaning from weight loss to experiencing the nature.

**Figure 1:**
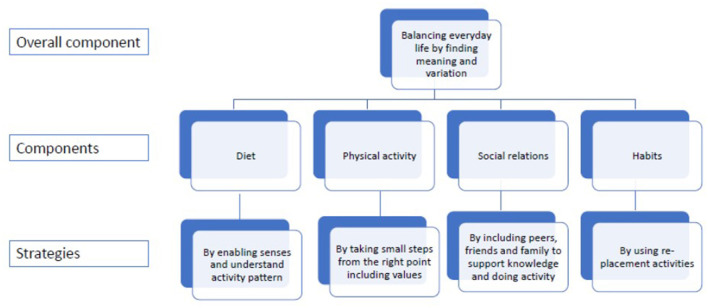
The relations between components and strategies.

For the citizens to find balance and understand the meaning and variation the activities in everyday life need to be in line with the person's resources of time, energy and ability (37). We suggest that the citizens need to identify barriers and opportunities to implement changes in line with their specific circumstances in life. Identifying barriers and opportunity related to activities could be addressed in all components presented her; diet, physical activities, social activities, and habits. We therefore recommend that balancing everyday life should be an overall component that need to be reached through the other four components and their strategies (Figure 1).

### Limitation of the Study

A limitation in the study was the number of citizens whish might have been influenced by two issues. The first issue being that the study was conducted at the university as it might be a barrier for some people who are not familiar with the higher education system and its institutions. The second issue was the fact that we did not use snowball principles as we had done when searching for the occupational therapists. However, as this study builds on our previously study with 34 individual interviews with citizens, we find this work to be valid. All the participants were women and even though knowledge from our previous study were found from both men and women the fact that only women discussed strategies might have influenced the results.

In relation to the participants in the RC it is a limitation that only 1 citizen, 2 occupational therapists and 1 researcher participated in all four meetings. However, to overcome this limitation every meeting started with a resume and a discussion based on the previous meeting to obtain the perspectives from the once who had not been represented in the previous meeting.

The fact that we had to conduct two Zoom meetings because of COVIDE-19 might be a limitation too. Conducting workshops online is challenging and even though we used discussion rooms provided by Zoom, the researchers' role became more like an interviewer rather than participants in the discussion. Despite this, we still describe this study as RC methodology because we met four times and the two first was done face-to-face indicating that the participants knew the procedure for RC methodology.

## Conclusion

Strategies assisting citizens with obesity to embrace to the five components of a holistic and sustainable weight loss program, should include utilizing the senses—such as taste, smell, texture and vision (colour)—when preparing and enjoying a meal; to be conscious of the patterns of activities surrounding the meal; to take one step at a time, not being overwhelmed; to find value in activities; to build self-confidence together with others and find common ground through participating in activity with family and friends and find re-placement activities. All of these strategies are related to everyday activities and participations, and we therefore suggest that the component of balancing everyday life through variation and meaning should be an overall component to provide encouraging support in day-to-day activities to stay healthy and reach weight loss goals.

## Data Availability Statement

The raw data supporting the conclusions of this article will be made available by the authors, without undue reservation.

## Ethics Statement

Ethical review and approval was not required for the study on human participants in accordance with the local legislation and institutional requirements. The patients/participants provided their written informed consent to participate in this study.

## Author Contributions

CJ-W and JRC designed the study and wrote the first draft. CJ-W, KL, and SS were involved in the data analysis. All authors were involved in interpretation of the findings, involved in the drafting the manuscript, read and revised subsequent drafts, and approved the final manuscript.

## Conflict of Interest

The authors declare that the research was conducted in the absence of any commercial or financial relationships that could be construed as a potential conflict of interest.

## Publisher's Note

All claims expressed in this article are solely those of the authors and do not necessarily represent those of their affiliated organizations, or those of the publisher, the editors and the reviewers. Any product that may be evaluated in this article, or claim that may be made by its manufacturer, is not guaranteed or endorsed by the publisher.
